# Sex-specific consequences of an induced immune response on reproduction in a moth

**DOI:** 10.1186/s12862-015-0562-3

**Published:** 2015-12-16

**Authors:** Andrea Barthel, Heike Staudacher, Antje Schmaltz, David G. Heckel, Astrid T. Groot

**Affiliations:** Department of Entomology, Max Planck Institute for Chemical Ecology, Hans-Knöll-Straße 8, 07745 Jena, Germany; University of Amsterdam, IBED, Science Park 904, 1098 XH Amsterdam, The Netherlands

**Keywords:** Life history trait, Immunity, Sexual communication, Reproduction, *Heliothis virescens*

## Abstract

**Background:**

Immune response induction benefits insects in combatting infection by pathogens. However, organisms have a limited amount of resources available and face the dilemma of partitioning resources between immunity and other life-history traits. Since males and females differ in their life histories, sex-specific resource investment strategies to achieve an optimal immune response following an infection can be expected. We investigated immune response induction of females and males of *Heliothis virescens* in response to the entomopathogenic bacterium *Serratia entomophila*, and its effects on mating success and the female sexual signal.

**Results:**

We found that females had higher expression levels of immune-related genes after bacterial challenge than males. However, males maintained a higher baseline expression of immune-related genes than females. The increased investment in immunity of female moths was negatively correlated with mating success and the female sexual signal. Male mating success was unaffected by bacterial challenge.

**Conclusions:**

Our results show that the sexes differed in their investment strategies: females invested in immune defense after a bacterial challenge, indicating facultative immune deployment, whereas males had higher baseline immunity than females, indicating immune maintenance. Interestingly, these differences in investment were reflected in the mate choice assays. As female moths are the sexual signallers, females need to invest resources in their attractiveness. However, female moths appeared to invest in immunity at the cost of reproductive effort.

**Electronic supplementary material:**

The online version of this article (doi:10.1186/s12862-015-0562-3) contains supplementary material, which is available to authorized users.

## Background

In ecological immunology, mounting a robust immune response against pathogens is considered a necessary but costly trait in terms of energy and resources. The effectiveness of the immune response thus depends on access to limited resources and on investment required by other costly life history traits, such as reproduction [1–5]. Many studies on the relationship between immunity and reproduction have attempted to link variation in immune responses with variation in reproductive success [5–8]. There is evidence that trade-offs exists between the two [9, 10]. For example, in *Drosophila melanogaster*, an increased sexual activity reduced the ability of the immune system to eliminate bacteria [9]. However, other studies reported a positive correlation between immune response and reproduction [11–13]. For example, mated females of *Gryllus texensis* are less susceptible to *S. marcescens* infection than virgins, suggesting that immune function and reproduction are positively correlated in these crickets [14]. Furthermore, a trade-off may not be present in general when two fitness-related traits do not share important resources, or if the resources are not limiting [13, 15].

Since males and females differ in their life histories, sex-specific resource investment strategies to achieve an optimal immune response following an infection can be expected [3, 16, 17]. In invertebrate species, males have been found to invest fewer resources in immune response than females [10, 18–20]. These sex-specific differences in immunity have been related to Bateman’s principle, i.e. females gain fitness by maximizing their lifespan through immunity investment, assuming that higher immunity increases longevity, which in turn increases the time for egg production and oviposition [17]. Males gain fitness by increasing their mating frequency, and should thus invest in increasing their mating rates instead of immunity [1, 17, 21–23]. Bateman’s principle is based on the generalization that females make a large investment into offspring because they have large immobile gametes, whereas males make a small investment into offspring because they produce small and mobile gametes [21]. However, Bateman’s principle as well as its consequences for investment in immune defense activation was shown to be reversed in species in which sex-roles are reversed and males invest more into offspring than females, e.g. by investing into parental care [23, 24].

As in other Lepidoptera, both *Heliothis virescens* females and males invest substantially into their offspring [25–27]. Female moths spend energetic and nutritional resources into the production of up to 1500 eggs, whereas males invest resources to produce a spermatophore, comprising up to 5 % of their body mass [25–27]. Males as well as females in *H. virescens* can mate multiple times in their lifetime, which is about 30 days in the laboratory at 25 °C [26–30]. However, unlike many animal species in which males can mate multiple times per day every day, *H. virescens* males and females only mate at most once per night [27, 31, 32]. Consequently, the number of matings is not only limited in females, but also in males. Thus, the male’s capacity to increase its number of matings is tightly linked to male longevity, because every night means one more mating opportunity. Therefore, both *H. virescens* males and females can be expected to invest into immunity in response to an immune challenge.

Investment in immunity can be intimately linked to sexual attractiveness. In many species, the attractiveness of a potential mate is determined by the quality of a sexual signal [33–35]. Parasite-mediated sexual selection indicates that sexual signals honestly reflect the quality of the signaler to the receiver [36]. In this scenario, honest signals are costly and condition dependent, and animals in good condition are able to afford to produce a higher quality signal, whereas animals in poor condition cannot invest much in sexual signaling [2, 37, 38]. Most studies that have investigated the relationship between reproductive success and immune response used species where males are the sexual signalers, competing for the attention of choosy females, e.g. birds, crickets or wolf-spiders [8, 39–43]. In moths, however, females produce a sex pheromone to attract males from a distance, i.e. hundreds of meters up to kilometers [44–48]. In *H. virescens*, males have been shown to differentiate between sex pheromone blends that differ in their qualitative and/or quantitative composition [35, 48, 49]. Thus, female fitness in this species is likely also determined by quality of the female signal, because it is crucially important to attract males. Whether the cost of immunity is associated with the quality of female signals in moths has not been determined.

In moths, the female sex pheromone has been shown to be an important sexual selection signal: females with a signal that deviates from the population mean attract significantly fewer males, so that these signals seem to be under stabilizing selection [50–53]. However, only a few studies have considered the female sex pheromone as an honest signal that is costly to produce and plastic depending on the condition of the females [54, 55]. A decrease in sex pheromone production or quality following infection might provide a further indication that the female pheromone of *H. virescens* is indeed an honest signal that can indicate the quality of the female to the male.

In this study, we investigated Bateman’s principle and immunity as well as the correlation between immunity and mating success in both sexes of *H. virescens* and between immunity and sexual signaling in females. We adapted the predictions for Bateman’s principle and immunity to the life history of *H. virescens* as follows. We hypothesize that females and males invest similarly in immunity, because longevity is equally important to the fitness of both sexes, i.e. for females to increase the time to produce and lay eggs and for males to increase their number of matings. We therefore predict that (1) immune gene expression will be similarly induced in males and females in response to an immune challenge. We also hypothesize that sexual attractiveness and immunity compete for the same resource pool and thus are negatively linked. Therefore, we further predict that an induced immune response (2) elicits reduced mating success in both sexes and (3) negatively affects the female sexual signal and sexual signaling behavior.

## Methods

### Ethics statement

Eggs of the moth *Heliothis virescens* were collected on private property in North Carolina, USA and no sampling permissions were required. *Heliothis virescens* is not an endangered species in the USA and is not protected by law. No ethical approval was required to work with this species in our study.

### Insects and bacterial culture

*Heliothis virescens* (JEN2; collected in 1988 in Clayton, North Carolina) was reared in environmental chambers at 26 °C, 60 % humidity, with a reverse 16:8 h light–dark cycle (scotophase starting at 8 am and ending at 16 pm). Larvae of *H. virescens* were fed on artificial pinto bean diet [56]. Pupae were collected, separated by sex, and placed in cups individually. Adults were provided with a 10 % honey-water solution. One to four day old virgin adults were used in all experiments of this study.

To induce an immune response in the moths we used freeze-dried cells of the entomopathogenic bacterium *Serratia entomophila*. This strain was shown to be deadly for *H. virescens* larvae and to induce hemocyte apoptosis in *H. virescens* larvae in an earlier study [57]. *S. entomophila* was obtained from the Department of Bioorganic Chemistry (MPICE, Jena, Germany). Bacteria were grown at 30 °C and 250 rpm in Caso medium. Overnight cultures of *S. entomophila* were centrifuged and the resulting supernatant was discarded. The extracted bacterial pellet was frozen at −20 °C. Samples were then frozen and dried in a lyophilisator at −80 °C for 5 days to kill the bacteria. Lyophilized cells of *S. entomophila* were stored at −20 °C. Dead cells of the entomopathogen were used in this study to measure the effects of immune defense, because this eliminates the confounding effects of metabolism and dynamics of a living pathogen in the moth [15].

### Activation of the immune system response by bacterial challenge in adult moths

For all our experiments, we challenged the moths by injecting adult males and females with bacteria. Injection of bacteria into the abdominal cavity mimics that bacteria enter the body via wounds in the cuticle, and has been commonly used to measure molecules that are involved in the immune response [58]. By using injections, we ensured a defined and equal immune response induction in all adult moths throughout all experiments. To induce an immune response in adult moths in all experiments of this study, adult moths were injected with 4 μg/4 μl lyophilized cells of *S. entomophila* diluted in 1x phosphate buffered saline (PBS). As control treatments, adults were either injected with 4 μl pure PBS (referred to as PBS-injected or wounded) or were not injected at all (referred to as non-injected or control). All injections were conducted using a 10-μl Hamilton syringe and were performed at the onset of photophase, approximately 16–20 h before the start of the experiments.

### Effect of immune challenge on immune gene expression in females and males

To determine the level and extent of immune response induction in both sexes, we assessed the expression of immune-related genes in female and male adult moths. An induced immune response in insects includes the expression of genes encoding a variety of antimicrobial peptides such as lysozyme, gloverin and hemolin to combat infections [59]. Besides antimicrobial peptides, phenoloxidase (PO) activating enzymes and heat shock proteins (Hsp) are important compounds of an efficient immune response in insects [59–61]. For our study we measured the expression levels of five immune related genes, i.e. heat shock protein 70, phenoloxidase activating factor, lysozyme, hemolin and gloverin. These genes have been found to be differentially regulated between control and *S. entomophila*-injected *H. virescens* larvae in a preliminary microarray study (unpublished data). We evaluated the expression differences of these 5 genes between *S. entomophila*-injected, PBS-injected and non-injected moths. Injections were carried out as described above. RNA extraction started 3 to 4 h after the onset of scotophase (20 h after injections). Moths were flash-frozen in liquid nitrogen and stored at −80 °C until RNA extraction. For each treatment, three replicates of each five bodies were used. Total RNA extraction was performed using TRIzol® (Invitrogen) according to the manufacturer’s protocol. To ensure that the isolated RNA was free of genomic DNA we performed a DNase treatment by adding 10 μl of Turbo DNase buffer and 1 μl of Turbo DNase enzyme (Ambion). Samples were then incubated for 30 min at 37 °C. RNA was additionally cleaned with the RNeasy MinElute Cleanup-Kit (Qiagen). To check the quality and concentration of the total RNA, isolated RNA was measured by ultraviolet (UV) detection using NanoDrop ND-1000 (Thermo Scientific). First-strand cDNA was synthesized using VersoTM SYBRR Green 2-Step QRT-PCRKit Plus ROX Vial (Thermo Scientific, ABgene, UK) according to the manufacturer’s instructions, starting with 900 ng of total RNA. Quantitative real-time PCR analysis was performed on a Stratagene Mx3000P QPCR System. Reagents were purchased from ABgene (Thermo Scientific) and used according to the manufacturer’s specifications. The PCR reaction for comparative quantification was run at 95 °C for 15 min and 40 cycles at 95 °C for 15 s, 58 °C for 30 s and 72 °C for 30 s. All PCR reactions were performed in technical duplicates, using three biological replicates for each treatment. The efficiency of each primer pair was calculated using the software program LinRegPCR [62]. To evaluate gene expression, ribosomal protein S18 (RpS18), based on a sequence from an In-house database, was used as a reference. Gene expression is given as copy number per 1000 molecules RpS18. All primers used in this study are shown in Additional file 1: Table S1.

### Effect of immune-challenge on the mating success of females and males

To investigate the consequence of an induced immune response on mating success in *H. virescens* females and males, we conducted mate choice experiments. Climate and light conditions in the experimental room were the same as for the rearing, i.e. 26 °C, 60 % relative humidity, and a reverse 16:8 h light–dark cycle (lights off at 8 am). Experiments were conducted in square gauze cages (33 × 33 × 33 cm). Each cage contained three adult moths, one chooser and two potential mates of the opposite sex (referred to as potential mate 1 and 2). Mate choice experiments were conducted in six assays, three male and three female choice assays (Additional file 2: Table S2). In assay 1 and 4, one of the two potential mates in one cage was injected with *S. entomophila* to trigger an immune response, whereas the other potential mate was not injected. To test whether wounding alone could influence mating behaviour, we conducted assays 2, 3, 5 and 6 as controls. The choice in these assays was between *S. entomophila*-injected and wounded potential partners (assay 2 and 5) or between wounded and control potential partners (assay 3 and 6). Choosers were not injected in any of the assays (1–6). The different mate choice experiments were randomly spread over several days, and at least two different assays were conducted on a single day, to avoid block or day effects. Fifty cages were observed in one night. To distinguish between the two potential mates in one cage, one was marked with a waterproof black marker, which was alternated between the two differentially treated potential mates to exclude bias due to marking. The moths were placed in the cages at the end of the photophase and 16 h after injection. Experiments started 150 min after the start of the scotophase, because females and males start to be reproductively active after ~2.5 h into scotophase [32, 63, 64]. All cages were checked every 30 min for copulation events until 450 min into scotophase, after which time point no further activity occurred. Mating behaviour was observed with the use of a red LED light (Sigma LED safety light). Only first matings in a cage were recorded.

To check whether mating behaviour was associated with the survival of adult moths for three days, we also recorded adult mortality daily for three days following the mate choice experiments. For these three days, all moths were kept individually in small plastic beakers (25 ml) and were provided with a 10 % honey-water solution.

#### Effect of immune-challenge on the female sexual signal

To assess the relationship between an induced immune response and the female sex pheromone profile, we injected virgin *H. virescens* with bacteria, with PBS, or we did not inject them, as described above, and analyzed their sex pheromone profile. Females for this experiment were not used in the mate choice experiments. We extracted sex pheromone glands 20 h after the injections (see above) when the virgin females were 2 days old, and between 3 and 4 h after the onset of scotophase. Glands were dissected with microscissors (FST instruments) and incubated for 30 min in conical vials containing 50 μl of hexane and 125 ng of the internal standard pentadecane to dissolve the sex pheromone. All pheromone samples were analyzed using a HP7890 gas chromatograph (GC) with a 7683 automatic injector. For the GC analysis, the hexane solution was evaporated with N_2_ to 2 μl. A volume of 4 μl (2 μl sample and 2 μl octane) was injected into a HP7890 gas chromatograph (GC) with a splitless inlet. The GC was equipped with a DB-WAXetr (extended temperature range) column of 30 m x 0.25 mm x 0.5 μm and was coupled with a flame ionization detector (FID). For further information on the GC analysis see Groot et al. (2010). Sex pheromone peaks were identified by comparing the retention times of our samples with the retention times of synthetic compounds (Pherobank, The Netherlands) of the sex pheromone blend of *H. virescens*. Pheromone peaks were integrated manually.

#### Effect of immune challenge on female signalling behaviour

To test whether the frequency and temporal pattern of female signalling, referred to as “calling”, was affected by induction of the immune response, we recorded the temporal patterns of calling behaviour of female moths. As the female sex pheromone is produced in a specialized gland that is located around the ovipositor [65], females were recorded as calling when the ovipositor with gland was clearly extruded from the female abdomen (see also Fig. 4). Injection experiments were done as described above. Two-day old virgin females were placed separately in transparent plastic cups (500 ml) at the end of the photophase (16 h after injection procedure). Calling behaviour experiments were conducted on three consecutive days. Each experimental day, females of all three treatments were tested to avoid block effects. In total, 178 females were observed, 34 at day one, 72 at day two and three, and females were used only once. As female sex pheromone is emitted at night, experiments started at the onset of the scotophase (at 8 am) and the experimental room was kept dark during the experimental period. Calling behaviour (yes or no) was recorded every five minutes until 450 min into scotophase, using a red light torch.

### Statistical analysis

Expression levels of immune-related genes were calculated relative to a housekeeping gene, the ribosomal protein S18 (RpS18), to adjust for possible differences among the samples which are not due to the treatments. Adjusted expression levels were then analyzed with one-way ANOVAs and consecutive Least-Squares means (LS-means) pairwise comparisons with Tukey adjustment. Data were log-transformed to obtain normality of residuals.

Female and male mate choice were tested with two-sided binomial tests. To statistically test the influence of immune system activation on the female sex pheromone composition, we calculated the relative percentage of each sex pheromone compound in the blend. Females that had a total sex pheromone amount of less than 25 ng were excluded from the analysis, because this was the threshold of accuracy of integration of the pheromone peaks. Moreover, due to a dirt peak in the GC run we had to exclude the minor compound Z7-16:Ald from our data set. Relative amounts of the compounds in the pheromone blend are not independent from each other. We therefore divided them by the minor compounds Z9-16:Ald to be able to perform multivariate analysis [35, 55]. We chose Z9-16:Ald as denominator, because this compound is not known to be relevant to attract males and did not differ between treatments in preliminary analyses. We then log10-transformed the data and performed a MANOVA analysis with the ratios of the five remaining compounds as response variable and treatment (*S. entomophila*-, PBS- or non-injected) as predictor variable. Since we detected an overall treatment effect in the MANOVA analysis (Pillai’s trace = 0.31, df = 2, *P* = 0.00035), we further analyzed the single compounds using ANOVA, followed by LS-means pairwise comparisons with Tukey adjustment. We additionally analyzed the ratio between the relative amounts of 16:Ald and Z11-16:Ald, because Z11-16:Ald is the major sex pheromone component and essential to attract males [35, 48] and the ratio 16:Ald/Z11-16:Ald was shown to have biological relevance to attract males as well: females with low ratios of 16:Ald to Z11-16:Ald were more attractive for males than females with high ratio [35]. To meet the assumption of normally distributed residuals, we log-transformed the ratio. We then conducted LS-means pairwise comparisons between our three treatments with Tukey adjustment for multiple comparisons with the ratio as response and treatment as predictor variable.

The influence of treatments on the calling behaviour of females was tested with a generalized linear mixed model using Poisson distribution in the glmmADMB package version 0.8.0 [66, 67] in the software R. To account for temporal autocorrelation, we summed up the calling events of individual females in blocks of 30 min, such that female’s calling in a period of 30 min were counted once for this period (even if they called more than one time in these 30 min), which resulted in a smooth parabolic curve (Fig. 4). We used calling as the response variable and treatment, time and time squared as fixed predictor variables. To account for repeated measurements over time, we added individual females as a random effect to the model, time was added as a random slope (time|individual female). Data were not overdispersed (sum of squared Pearson residuals/residuals degrees of freedom = 0.85). An interaction effect of time and treatment was tested but left out of the final model, because it was not significant. The overall significance of treatment was tested with a Chi-square test, using the car package. All statistical analyses were conducted in the program R, version 3.0.2 [68].

## Results

### Effect of immune challenge on immune gene expression in females and males

**Fig. 1:**
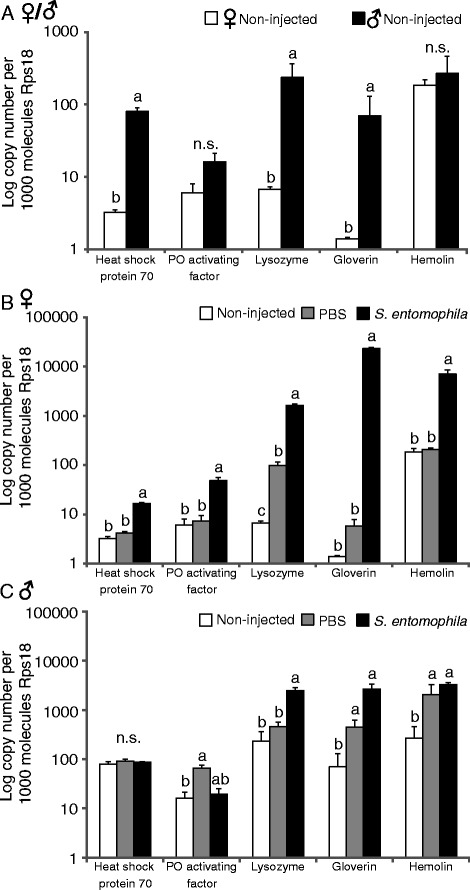
Expression level of immune-related genes in non-injected, *S. entomophila-* or PBS-injected *Heliothis virescens* females and males. Expression levels were compared between control females and males (**a**) and among all treatments in females (**b**) and males (**c**). Values are given as logarithmic copy number per 1000 molecules RpS18. Bars represent the mean of 3 biological replicates with corresponding standard errors. Different letters above the bars represent significant differences based on ANOVA and LS-means pairwise comparisons with Tukey adjustment

Males had higher baseline expression levels of immune-related genes than females (Fig. 1a). Hsp 70, lysozyme and gloverin exhibited significantly higher expression in control males than in control females, whereas PO activating factor and hemolin were similarly expressed in both sexes. When *S. entomophila* was injected into female and male moths, transcription levels of immune-related genes were induced in both sexes compared to the control (Fig. 1b and c). In females, a significant increase in transcript levels of all tested immune-related genes was observed after injection of *S. entomophila* compared to wounded and control females (Fig. 1b). In males, only lysozyme was significantly upregulated in males after injection with *S. entomophila* compared to wounded and control males (Fig. 1c)*.* Levels of gloverin and hemolin, which were significantly upregulated in males upon *S. entomophila* injection, were also upregulated in wounded males compared to control males. The transcript level of PO activating factor was significantly higher in wounded males compared to control males but not compared to *S. entomophila*-injected individuals. Overall, immune-related genes in females were induced only in response to bacterial injections, whereas in males immune-related genes were similarly induced after wounding and bacterial injections (see Additional file 3: Table S3 for significance values).

### Effects of immune challenge on the mating success of females and males

**Fig. 2:**
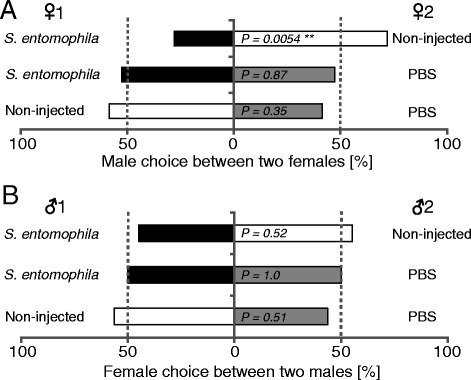
Effect of immune system activation on mating behaviour in *Heliothis virescens*. **a** Male choice in three independent two-choice mating experiments. **b** Female choice in three independent two-choice mating assays. Black colours correspond to *S. entomophila-*injected adults, grey colours correspond to PBS-injected adults and white colours to non-injected adults. Dashed lines indicate 50 % of the total mated adults (see Additional file 2: Table S2 for sample sizes). Significant differences are indicated by **P < 0.01, as tested with two-sided binomial tests

Males that had a choice between *S. entomophila*-injected and control females mated significantly more often with control females than with *S. entomophila-*injected females (*P* = 0.005) (Fig. 2a). Males that had a choice between *S. entomophila-*injected and wounded females or between control and wounded females did not mate significantly more with one type of female (Fig. 2a). In the female choice experiments, females mated similarly often with all types of males offered (Fig. 2b). We did not observe any adult mortality within three days after the mating experiment among all treatments.

#### Effect of immune challenge on the female sexual signal

**Fig. 3:**
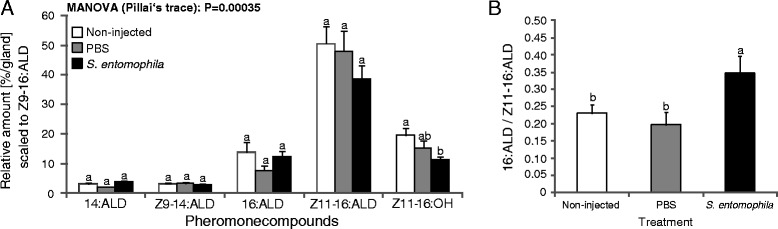
Influence of immune defense activation on sex pheromone composition of *Heliothis virescens* females. **a** Relative amounts of five compounds scaled to Z9-16:Ald, **b** Ratio between 16:Ald and Z11-16:Ald. Non-injected *n* = 38; PBS-injected *n* = 25*; S. entomophila*-injected *n* = 38. Different letters above the bars indicate significant differences between treatments at a level of alpha < 0.05 based on LS-means pairwise comparisons with Tukey adjustment for multiple comparisons

In female moths, the sex pheromone profile was affected by the immune challenge. Females injected with *S. entomophila* produced significantly lower amounts of Z11-16:OH than control females, but not less than wounded females (Fig. 3a). The relative amounts of 14:Ald, Z9-14:Ald, 16:Ald, Z11-16:Ald were not significantly different between the treatments (Fig. 3a and Additional file 4: Table S4A-C for significance values, Additional file 5: Figure S1). The ratio of 16:Ald to Z11-16:Ald was significantly higher in *S. entomophila*-injected females compared to wounded and control females (Fig. 3b).

#### Effect of immune-challenge on female signalling behaviour

**Fig. 4:**
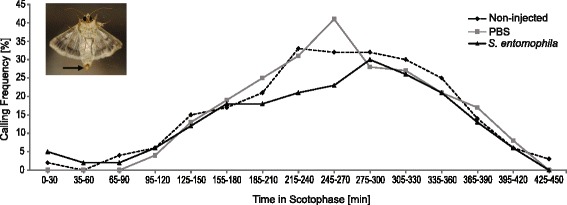
Calling activity of virgin *Heliothis virescens* females during scotophase. Individual calling behaviour was registered every 5 min, calling events of 30 min periods were grouped together. Calling behaviour is depicted as the percentage of females that called per time interval (non-injected *n* = 58; PBS-injected *n* = 60; *S. entomophila-*injected *n* = 60). The overall treatment effect was tested with a generalized linear mixed model (glmmADMB in R). Photo: calling *H. virescens* female, arrow: extruded ovipositor with sex pheromone gland

The number of females calling was not affected by the different treatments (*χ*^2^ = 4.85, df = 2, *P* = 0.088). We did not find an interaction effect of treatment and time, suggesting that calling timing was not affected by the immune challenge (Fig. 4). The calling behaviour of all females followed similar temporal and frequency patterns with an ‘on and off’ calling behaviour throughout scotophase. The majority of all females started calling 120 min into scotophase and stopped calling after 420 min, with a peak of calling activity between 240 and 360 min.

## Discussion

Our study revealed a negative correlation between immune system activation and reproduction in the moth *H. virescens* on a physiological as well as a behavioral level. However, in contrast to our hypothesis, we found that females and males invest differently in immunity: males had higher baseline immunity than females, indicating immune maintenance, while immune system activation was higher in females than in males in response to a bacterial challenge, indicating immune deployment. Our hypothesis that sexual attractiveness and immunity compete for the same resource pool and thus negatively affect each other was partly confirmed: mating success was reduced in *S. entomophila*-injected females, but not in *S. entomophila*-injected males. In addition, injection with *S. entomophila* caused females to modify their sex pheromone profile.

### Effect of immune challenge on immune gene expression in females and males

When assessing the immune status of organisms, it is important to distinguish between immune maintenance, which implies physiological costs to keep the immune system at a certain level of readiness, and immune deployment, which implies physiological costs of immune system activation to combat pathogens [69]. We tested this by measuring the expression level of immune-related genes in adults upon challenge with *S. entomophila*, and found that immune deployment was higher in *H. virescens* females than in males. This is in line with reports from other insects. For example, in the scorpionfly *Panorpa vulgaris*, females had a higher lysozyme-like activity and a higher number of (phagocytically active) hemocytes than males [70]. In the grasshopper *Melanoplus sanguinipes*, adult females showed higher PO activities than males in response to a fungus infection [71].

Interestingly, our immune gene study showed that immune maintenance was higher in males than in females. If such an investment increases their longevity, male fitness is likely to be increased: *Heliothis virescens* males only mate at most once per night, so that every additional night of being alive means another mating opportunity [26, 31]. Additionally, *H. virescens* males invest in their offspring by transferring resources for a spermatophore to the female, which can make up to 5 % of their body weight and contains not only sperm, but can also contain one third of the male’s zinc supplies as well as sugars and proteins [27, 72]. Male investment into offspring has been shown to affect their investment in immunity in various species. For instance, in the pipefish *Syngnathus typhle*, a sex role reversed species where males invest more in offspring than females, males showed higher innate and adaptive immunity than females [23]. In another species with sex role reversal, the spider *Allocosa brasiliensis*, males showed a higher encapsulation response than females in response to a nylon filament implant [24]. Interestingly, in *H. virescens*, which neither shows conventional sex roles nor sex role reversal but an investment of both sexes into offspring, we found that both sexes invested into immunity in different ways: males had higher immune maintenance, while females showed higher immune deployment.

The immune system of *H. virescens* females also seemed more specific than that of males, because immune gene expression levels differed more between wounding and bacterial challenge in females than in males (Fig. 1). To save resources, it is likely important for animals to distinguish between wounding and bacterial challenge, because the activation of an immune response is costly and can negatively affect other life-history traits [3, 73, 74]. This negative correlation may affect female fitness more than male fitness in *H. virescens,* because females are the sexual signalers, whereas males have been shown to be choosy [35, 48]. Therefore, access to males is important for female fitness, and females likely need to invest resources in their attractiveness [54, 55]. Consequently, females may have evolved a more specific immune response than males, which is only activated in response to cues of pathogens, whereas males may afford constitutively high levels of immune defense and react stronger to minor cues of disease, like wounding.

### Effect of immune challenge on mating success of females and males

Since behavior is intimately linked to the physiology of insects, we hypothesized that sexual attractiveness and immunity compete for the same resource pool and thus negatively affect each other. In the mate choice experiments, we found male choice but not female choice. Specifically, we found in the male choice experiment that *H. virescens* males mated less with *S. entomophila*-injected females and chose more control females in a two-choice test. Males thus seem to choose the healthier female, as would be expected by parasite-mediated sexual selection theory, which states that individuals should prefer parasite-free mates because the genes for resistance would be transmitted to their offspring [5, 36]. Additionally, the transmission avoidance hypothesis postulates that individuals should avoid parasitized mates to avoid getting infected themselves [5, 75, 76]. Furthermore, the resource provisioning model assumes that parasite-free individuals should be better at providing resources to mates and their offspring [5, 75]. Therefore, males of *H. virescens* may choose healthier females to either avoid getting infected themselves or ensure that their offspring are provided with optimal resources.

However, we also found that males did not distinguish between wounded females and *S. entomophila*-injected females or wounded females and control females. This may be explained by comparing expression levels in our immune gene expression assays, where we found that wounding also induced an immune response in females of *H. virescens*, e.g. in the expression of lysozyme. Additionally, the differences in immune response induction between control and wounded, or wounded and *S. entomophila*-injected females were not as big as between control and *S. entomophila*-injected females (Fig. 1b). These smaller differences might not have been enough to be recognized by the males. Furthermore, it is known that stress factors, like wounding, induce increases in octopamine which leads to changes in the level of juvenile hormone and thus can inhibit reproduction [77, 78]. This phenomenon may explain why males do not distinguish between wounded females and *S. entomophila*-injected females, since males may detect a stress response in both types of females and thus these females appear to be similar.

We can exclude the possibility that male choice was due to an acute risk of *S. entomophila*-injected females to die (in the course of three days), because in all three treatments females survived for more than three days.

In the female mate choice experiments, all types of males were chosen similarly often, indicating that females did not distinguish between differentially treated males. As males reacted less, physiologically, to the bacterial challenge than females, in terms of up-regulation of immune-related genes, it is likely that all males appeared similar to the females in the female mate-choice experiments. In addition, oxidative stress generated by immune system activation may cause a negative relationship between immunity and attractiveness [5, 79, 80]. Since the immune deployment was lower in *H. virescens* males than females, males may suffer less oxidative damage and thus appear healthy and still attractive for females [79]. Furthermore, heat shock proteins (HSPs) were found to be upregulated under stress conditions in insects [81, 82]. Our results that males do not upregulate HSP 70 in response to *S. entomophila*, suggest that these males may not be stressed and might thus explain why females are not able to differentiate between healthy and sick males in our mate choice experiments. On the other hand, females were found to upregulate the expression of HSP70 in response to *S. entomophila*. Males may recognize the stress response in infected females and thus avoid sick females, as was found in our mate choice experiment.

In *Tenebrio molitor* juvenile hormone (JH) was found to induce attractiveness, while suppressing the expression of phenoloxidase [10]. Our finding that the expression level of a phenoloxidase activating factor remains constant in males, but significantly increases in females in response to injection with *S. entomophila,* suggests that immunity is negatively correlated with reproduction in females, but not in males. Another explanation for our results might be that the pathogen applied in our study does not cause enough harm in males for choosy females to benefit by selecting males with genes for parasite resistance or the ability to provide optimal resources for their offspring [5].

### Effect of immune challenge on sexual signal and calling behaviour in females

We found the immune system activation in *H. virescens* females to be associated with an altered sex pheromone profile. The ratio of 16:Ald to Z11-16:Ald was significantly higher in *S. entomophila*-injected females than in wounded or control females. Z11-16:Ald is the major sex pheromone component in the *H. virescens* sex pheromone blend and is essential to attract males [35, 48]. As Groot et al. (2014) showed that females with higher ratios of 16:Ald/Z11-16:Ald (and thus less of the major component) were less attractive to males in the field than females with lower ratios [35], these results indicate that infected females likely attract fewer males than healthy females under field conditions. Our results are consistent with studies by Worden et al. (2000), who found that an immune challenge reduces the attractiveness and reproductive success in grain beetles [83]. Therefore, our findings corroborate the hypothesis that production of pheromones is a condition dependent sexual trait [38, 84].

Interestingly, the changes in the sexual signal towards a less attractive profile did not coincide with a change in calling behavior. These results indicate that female immunity and only the quality of the female sexual signal are linked, and suggest that the female sex pheromone in moths is an honest signal that indicates the quality of a female to the male, which also fits the framework of parasite mediated sexual selection theory [36].

Since the pheromone profile differed between wounded and *S. entomophila*-injected females, while males did not mate more with wounded than with *S. entomophila*-injected females in the mate choice experiments, the altered pheromone profile cannot fully explain our mate choice results. Most likely, at close range in mating cages, other traits than the female long-range sex pheromone are important for male choice and female mating success. In moths, males produce and emit a courtship pheromone that is likely important for female choice [85, 86]. However, a male courtship pheromone cannot explain male choice for specific females. Perhaps cuticular hydrocarbon profile (CHCs) plays a role in mate choice at close range in *H. virescens*, as has been found in many insect species, e.g. *Drosophila*, crickets and beetles [87–89]. CHCs have hardly been investigated in moths, with a few exceptions [90, 91] and their role in moth sexual communication is still unknown.

In this study we found evidence for a negative correlation between the expression of immune-related genes and mating success as well as the female sexual signalling. This negative correlation between the two traits strongly suggests that there is a trade-off between immunity and reproduction in *H. virescens* females. However, testing theories relating to trade-offs between reproduction and immunity require large-scale experiments that assay immune parameters and reproductive processes in the same individuals, while we tested different groups of moths in our experiment.

## Conclusion

In the noctuid moth *H. virescens,* we found that investment in immunity is linked to the life history trajectories of males and females*,* and can negatively impact sexual attractiveness and reproduction. Immune response is important for both sexes in *H. virescens*, because both females and males mate only once per night and invest substantially into their offspring. Accordingly, in our immune gene expression assays, we found that both males and females invested in immunity. However, the sexes differed in their investment strategies: females invested in immune defense after a bacterial challenge, whereas males had higher baseline immunity than females. Interestingly, these differences in investment were reflected in the mate choice assays. Males chose more for non-injected females than for *S. entomophila*-injected females, whereas females did not show a preference for differentially treated males. Furthermore, *S. entomophila*-injected females had an altered sexual signal compared to non-injected females. Thus, there is a negative correlation between immune system activation and reproduction in females. We did not find this pattern in males, probably because *H. virescens* males invest in immunity maintenance. It will be interesting to determine whether these sex-specific differences in the type of immunity, i.e. immune deployment in females and immune maintenance in males are a general phenomenon.

### Availability of supporting data

The data sets supporting the results of this article will be available in the Dryad.org repository (upon acceptance of the manuscript).

## Additional files

Additional file 1: Table S1. Primer sequences used in qRT-PCR analysis. (PDF 9 kb) Additional file 2: Table S2. Experimental setup for mate choice experiments. (PDF 31 kb) Additional file 3: Table S3. Pairwise comparisons (LS-means with Tukey adjustment) of the expression level of immune-related genes between all treatments. Male and female moths were injected with *Serratia entomophila* (SER), PBS (PBS) or non-injected (NON); *n* = 3 for all groups. The overall treatment effect was tested by one-way-ANOVA with *P* < 0.001 for all genes. (PDF 32 kb) Additional file 4: Table S4. A. Overall treatment effect for differences in *Heliothis virescens* female sex pheromone between *Serratia entomophila*-injected (*n* = 38), non-injected (*n* = 38) and PBS- injected (*n* = 25) females. Table S4B. Effect of treatment on individual compounds of *Heliothis virescens* female sex pheromone tested with ANOVAs and LS-means pairwise comparisons (with Tukey adjustment) for compounds with treatment effect of *P* < 0.05. SER: *Serratia entomophila-*injected (*n* = 38), NON: non-injected (*n* = 38), PBS: PBS-injected (*N* = 25). Table S4C. 16:ALD/Z11-16:ALD ratio in *Heliothis virescens* female sex pheromone. ANOVA for overall treatment effect and LS-means pairwise comparisons with Tukey adjustment between females of different treatment groups, SER: *Serratia entomophila-* injected (*n* = 38), NON: non-injected (*n* = 38), PBS: PBS-injected (*n* = 25). (PDF 18 kb) Additional file 5: Figure S1. Pheromone composition of *Heliothis virescens* females that were non-injected (*n* = 38), injected with *S. entomophila* (*n* = 38) or with PBS (*n* = 25). Z7-16:Ald was excluded from the analysis, see main text for explanation. (PDF 32 kb)
