# Supplementary material for: Sex-specific consequences of an induced immune response on reproduction in a moth
Source: BMC Evol Biol. 2015 Dec 16;15:282. doi: 10.1186/s12862-015-0562-3 (PMC4681174; doi:10.1186/s12862-015-0562-3)
Supplement: Additional file 1: Table S1. — Primer sequences used in qRT-PCR analysis. (PDF 9 kb) [file 12862_2015_562_MOESM1_ESM.pdf]

Table S1. Primer sequences used in qRT-PCR analysis

Gene name	Accession number	Forward primer (5’-3’)	Reverse primer (5’-3’)
Heat shock protein 70	ACV32640	GTGCTCAGGATCATCAACGA	AGGTCGAAGATGAGCACGTT
PO activating factor	ACI32835	TGATCAGCCACAGCGTAAAG	CTCCGAATTTGGTCTCTCCA
Lysozyme	AAD00078	CGCTAGAAAGACGGACAAGG	CATTTCAGCGCAAGTGACAT
Gloverin	ACR78446	AGCAGCTTCTTGGGAGGAC	TCCTTATGGACATCAAGAGCAG
Hemolin	ACC91897	TTCCTGAGCCGAAGAATCAC	AATGTTCAGCCAACACCACA
Ribosomal protein S18 (RpS18)	In-house database	GCGTGCTGGAGAATGTACTG	GCCTGTTGAGGAACCAGTCT
