# Supplementary material for: Sex-specific consequences of an induced immune response on reproduction in a moth
Source: BMC Evol Biol. 2015 Dec 16;15:282. doi: 10.1186/s12862-015-0562-3 (PMC4681174; doi:10.1186/s12862-015-0562-3)
Supplement: Additional file 2: Table S2. — Experimental setup for mate choice experiments. (PDF 31 kb) [file 12862_2015_562_MOESM2_ESM.pdf]

Table S2. Experimental setup for mate choice experiments.

Assay		Chooser	Potential mate 1	Potential mate 2	Sample size [n]
Male choice	1	♂ Non-injected[1]	♀ *S. entomophila*[2]	♀ Non-injected[1]	43
	2	♂ Non-injected[1]	♀ *S. entomophila*[2]	♀ PBS[3]	38
	3	♂ Non-injected[1]	♀ Non-injected[1]	♀ PBS[3]	41
Female choice	4	♀ Non-injected[1]	♂ *S. entomophila*[2]	♂Non-injected[1]	58
	5	♀ Non-injected[1]	♂ *S. entomophila*[2]	♂ PBS[3]	48
	6	♀ Non-injected[1]	♂ Non-injected[1]	♂ PBS[3]	39

[1]Referred to as control individuals; [2]Referred to as infected individuals; [3]Referred to as wounded individuals
