# Supplementary material for: Sex-specific consequences of an induced immune response on reproduction in a moth
Source: BMC Evol Biol. 2015 Dec 16;15:282. doi: 10.1186/s12862-015-0562-3 (PMC4681174; doi:10.1186/s12862-015-0562-3)
Supplement: Additional file 3: Table S3. — Pairwise comparisons (LS-means with Tukey adjustment) of the expression level of immune-related genes between all treatments. Male and female moths were injected with Serratia entomophila (SER), PBS (PBS) or non-injected (NON); n = 3 for all groups. The overall treatment effect was tested by one-way-ANOVA with P < 0.001 for all genes. (PDF 32 kb) [file 12862_2015_562_MOESM3_ESM.pdf]

Table S3. Pairwise comparisons (LS-means with Tukey adjustment) of the expression level of immune-related genes between all treatments. Male and female moths were injected with *Serratia entomophila* (SER), PBS (PBS) or non-injected (NON); n=3 for all groups. The overall treatment effect was tested by one-way-ANOVA with P < 0.001 for all genes.

Gene	Sex	Comparison	df	t-ratio	P-Value
**Heat shock protein 70**	♀	PBS – NON	12	-2.125	0.33
		SER – PBS	12	-11.837	**<0.001*** **
		SER – NON	12	-13.963	**<0.001*** **
	♂	PBS – NON	12	-1.310	0.77
		SER – PBS	12	0.477	1.00
		SER – NON	12	-0.833	0.96
	♀ vs ♂	NON-NON	12	-27.368	**<0.001*** **
**Gloverin**	♀	PBS – NON	12	-1.693	0.56
		SER – PBS	12	-11.711	**<0.001*** **
		SER – NON	12	-13.404	**<0.001*** **
	♂	PBS – NON	12	-3.601	**0.03***
		SER – PBS	12	-2.652	0.16
		SER – NON	12	-6.253	**<0.001*** **
	♀ vs ♂	NON-NON	12	-4.060	**0.02***
**Hemolin**	♀	PBS – NON	12	-0.297	1.00
		SER – PBS	12	-6.106	**<0.001*** **
		SER – NON	12	-6.403	**<0.001*** **
	♂	PBS – NON	12	-3.988	**0.02***
		SER – PBS	12	-1.371	0.74
		SER – NON	12	-5.359	**0.002****
	♀ vs ♂	NON-NON	12	0.245	1.00
**Lysozyme**	♀	PBS – NON	12	-7.291	**<0.001 *****
		SER – PBS	12	-7.858	**<0.001 *****
		SER – NON	12	-15.149	**<0.001 *****
	♂	PBS – NON	12	-2.523	0.19
		SER – PBS	12	-4.752	**0.005****
		SER – NON	12	-7.275	**<0.001 *****
	♀ vs ♂	NON-NON	12	-9.007	**<0.001 *****
**Phenoloxidase activating factor**	♀	PBS – NON	12	-0.686	0.98
		SER – PBS	12	-4.609	**0.006****
		SER – NON	12	-5.294	**0.002 ****
	♂	PBS – NON	12	-3.505	**0.04***
		SER – PBS	12	3.107	0.08
		SER – NON	12	-0.398	1.00
	♀ vs ♂	NON-NON	12	-2.547	0.19
