# Supplementary material for: Sex-specific consequences of an induced immune response on reproduction in a moth
Source: BMC Evol Biol. 2015 Dec 16;15:282. doi: 10.1186/s12862-015-0562-3 (PMC4681174; doi:10.1186/s12862-015-0562-3)
Supplement: Additional file 4: Table S4. — A. Overall treatment effect for differences in Heliothis virescens female sex pheromone between Serratia entomophila-injected (n = 38), non-injected (n = 38) and PBS- injected (n = 25) females. Table S4B. Effect of treatment on individual compounds of Heliothis virescens female sex pheromone tested with ANOVAs and LS-means pairwise comparisons (with Tukey adjustment) for compounds with treatment effect of P < 0.05. SER: Serratia entomophila-injected (n = 38), NON: non-injected (n = 38), PBS: PBS-injected (N = 25). Table S4C. 16:ALD/Z11-16:ALD ratio in Heliothis virescens female sex pheromone. ANOVA for overall treatment effect and LS-means pairwise comparisons with Tukey adjustment between females of different treatment groups, SER: Serratia entomophila- injected (n = 38), NON: non-injected (n = 38), PBS: PBS-injected (n = 25). (PDF 18 kb) [file 12862_2015_562_MOESM4_ESM.pdf]

Table S4A. Overall treatment effect for differences in *Heliothis virescens* female sex pheromone between *Serratia entomophila* injected (n=38), non-injected (n=38) and PBS-injected (n=25) females.

MANOVA	df (degree of freedom)	Pillai's trace	Approximate F	Pr(>F)
Treatment	2	0.31	3.45	0.00035
Residuals	98			

Table S4B. Effect of treatment on individual compounds of *Heliothis virescens* female sex pheromone tested with ANOVAs and LS-means pairwise comparisons (with Tukey adjustment) for compounds with treatment effect of P < 0.05. SER: *Serratia entomophila*-injected (n=38), NON: non-injected (n=38), PBS: PBS-injected (N=25)

**ANOVAs, individual compounds**				**LS means pairwise comparisons (Tukey adjusted)**			
Compound	df	F value	Pr(>F)	Comparison	df	t-ratio	P-value
				SER-NON		-1.40	0.35
14:ALD	2	2.08	0.13	SER-PBS	98	-1.95	0.13
				PBS-NON		0.71	0.76
				SER-NON		0.97	0.60
Z9:14:ALD	2	0.61	0.55	SER-PBS	98	0.91	0.64
				PBS-NON		-0.05	0.64
				SER-NON		-0.14	0.99
16:ALD	2	1.05	0.36	SER-PBS	98	-1.36	0.37
				PBS-NON		1.23	0.44
				SER-NON		1.67	0.22
Z11-16:ALD	2	1.47	0.24	SER-PBS	98	1.08	0.53
				PBS-NON		0.40	0.91
				SER-NON		2.82	**0.016***
Z11-16:OH	2	4.0	**0.021***	SER-PBS	98	1.47	0.31
				PBS-NON		1.04	0.55

Table S4C. 16:ALD /Z11-16:ALD ratio in *Heliothis virescens* female sex pheromone. ANOVA for overall treatment effect and LS-means pairwise comparisons with Tukey adjustment between females of different treatment groups, SER: *Serratia entomophila*-injected (n=38), NON: non-injected (n=38), PBS: PBS-injected (n=25).

				**LS means pairwise comparisons (Tukey adjusted)**			
**Tested Ratio**	**df**	**F-value**	**Pr(>F)**	**Comparison**	**df**	**t-ratio**	**P-value**
				SER-NON		2.45	**0.042***
16:ALD / Z11-16:ALD	2	6.74	**0.0018****	SER-PBS	98	3.54	**0.018****
				PBS-NON		1.36	0.37
