# Supplementary material for: Sex-specific consequences of an induced immune response on reproduction in a moth
Source: BMC Evol Biol. 2015 Dec 16;15:282. doi: 10.1186/s12862-015-0562-3 (PMC4681174; doi:10.1186/s12862-015-0562-3)
Supplement: Additional file 5: Figure S1. — Pheromone composition of Heliothis virescens females that were non-injected (n = 38), injected with S. entomophila (n = 38) or with PBS (n = 25). Z7-16:Ald was excluded from the analysis, see main text for explanation. (PDF 32 kb) [file 12862_2015_562_MOESM5_ESM.pdf]

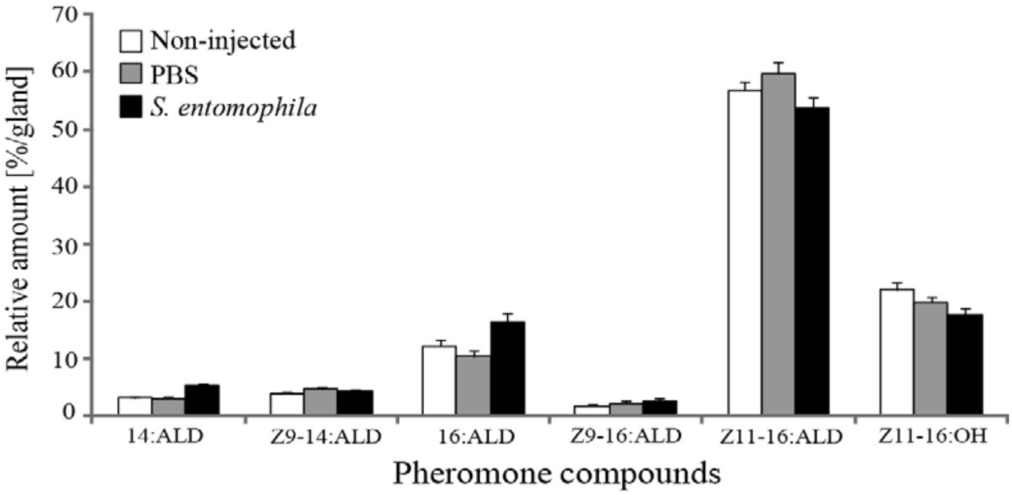

Figure S1: Pheromone composition of *Heliothis virescens* that were non-injected (n=38), injected with *S. entomophila* (n=38) or with PBS (n=25). Z7-16:Ald was excluded from the analysis, see main text for explanation**.**
